# Low carbohydrate diet prevents Mcl-1-mediated resistance to BH3-mimetics

**DOI:** 10.18632/oncotarget.12309

**Published:** 2016-09-28

**Authors:** Camila Rubio-Patiño, Jozef P. Bossowski, Elodie Villa, Laura Mondragón, Barbara Zunino, Emma Proïcs, Johanna Chiche, Frédéric Bost, Els Verhoeyen, Jean-Ehrland Ricci

**Affiliations:** ^1^ Inserm, U1065, Centre Méditerranéen de Médecine Moléculaire (C3M), Équipe “Contrôle Métabolique des Morts Cellulaires”, Nice, France; ^2^ Université Nice Côte d'Azur, Inserm, C3M, France; ^3^ Centre Hospitalier Universitaire de Nice, Département d'Anesthésie Réanimation, Nice, France; ^4^ Inserm, U1065, Centre Méditerranéen de Médecine Moléculaire (C3M), Équipe “Cellular and Molecular Physiopathology of Obesity and Diabetes”, Nice, France

**Keywords:** metabolism, cancer, resistance to treatment, Mcl-1, low carbohydrate diet

## Abstract

Overexpression of Mcl-1 is implicated in resistance of several cancers to chemotherapeutic treatment, therefore identifying a safe way to decrease its expression in tumor cells represents a central goal. We investigated if a modulation of the diet could impact on Mcl-1 expression using a Myc-driven lymphoma model. We established that a partial reduction of caloric intake by 25% represents an efficient way to decrease Mcl-1 expression in tumor cells. Furthermore, using isocaloric custom diets, we observed that carbohydrates (CHO) are the main regulators of Mcl-1 expression within the food. Indeed, feeding lymphoma-bearing mice with a diet having 25% less carbohydrates was sufficient to decrease Mcl-1 expression by 50% in lymphoma cells. We showed that a low CHO diet resulted in AMPK activation and mTOR inhibition leading to eukaryotic elongation factor 2 (eEF2) inhibition, blocking protein translation elongation. Strikingly, a low CHO diet was sufficient to sensitize Myc-driven lymphoma-bearing mice to ABT-737-induced cell death *in vivo*. Thus reducing carbohydrate intake may represent a safe way to decrease Mcl-1 expression and to sensitize tumor cells to anti-cancer therapeutics.

## INTRODUCTION

While extremely diverse in origin and in the type of associated mutations, cancer cells share common features, including the ability to use diverse sources of energy for cell proliferation and the ability to escape cell death [1]. Otto Warburg, in the 1920′s, was one of the first to describe that cancer cells have a special metabolism, as they are avidly relying on glucose to produce energy [2]. This metabolic phenomenon is now referred to as the “Warburg effect”. Further research established that the Warburg effect, observed in about 80% of cancers, is not only required for energy production but also for the generation of macronutrients and the redox systems that are required for the rapid proliferation of cancer cells [3]. Since this discovery, researchers and pharmaceutical companies are developing ways to modulate metabolism in order to limit tumor appearance and/or enhance treatment. One central way to control the metabolism of tumor cells is to modify the whole metabolism of the body through the modulation of food intake. Indeed, it has been known for more than a century that low caloric intake is associated with a reduced risk of several human diseases including cancer, while excess caloric intake is associated with higher cancer risk and shortened lifespan [4]. It is now clear that lowering caloric intake in general or specific macronutrients can prolong life span and improve health in a broad range of organisms when compared with unrestricted food intake. Overtime it was suggested that diet-induced regulation of insulin and its closely related hormone, Insulin-like growth factor 1 (IGF-1), were main events involved in protection against cancer incidence [4, 5]. However how food intake and how specific macronutrients impact on the response to chemotherapy is still largely unknown.

According to the International Agency for Research on Cancer (IARC), the worldwide age standardized incidence rate of non-Hodgkin lymphoma (NHL) among both sexes is estimated at 5 per 100,000 people, and is considered among the fastest rising cancer both in frequency and death rates in the United States. NHL includes a heterogeneous variety of malignancies of which the vast majority derives from B lymphocytes. Overexpression of the antiapoptotic members of the Bcl-2 family is one of the best-characterized alterations associated with NHL. Bcl-2 family members are primary regulators of mitochondrial integrity. Indeed, upon activation, Bax and Bak will induce the mitochondrial outer membrane permeabilization (MOMP) resulting in the release of inter-membrane space proteins including cytochrome c. Once released, cytochrome c will bind Apaf-1 and caspase-9 leading to apoptosome formation, caspase activation and cell death. Bax, Bak and possibility Bok are considered to be absolutely required for MOMP and for the apoptotic mitochondrial pathway as their removal leads to resistance to a variety of stimuli [6–8]. The activity of these proapoptotic proteins is tightly controlled by either the anti-apoptotic Bcl-2 family members (Bcl-2, Bcl-xL, Bcl-w, Mcl-1, Bcl-B and A1), which restrain the induction of cell death, thus promoting cellular survival, or by the proapoptotic BH3-only proteins (Bim, Puma, Noxa, Bad, Bmf, Bid, Bik and Hrk) [9], which actively participate in inducing cell death.

Anti-apoptotic Bcl-2 family members function by directly binding BH3-only molecules as well as pro-apoptotic effectors. This notion was critical to the design of therapeutic strategies that have been developed to inhibit the prosurvival members. These include BH3 mimetics, such as ABT-737 and ABT-263 (navitoclax) [10, 11], which bind with high affinity to Bcl-2 and Bcl-xL resulting in Bax and Bak-dependent apoptosis. While efficient in some settings, the low affinity of these compounds for Mcl-1 or A1 limits their use in cells presenting high endogenous levels of Mcl-1 expression, such as aggressive forms of NHL. Mcl-1 has the particularity to have a short half-life and to be highly regulated at transcriptional, translational and post-translational levels [12]. Additionally, Mcl-1 expression is tightly associated with cell metabolism [13]. In fact, it has been shown that a shorter form of Mcl-1 could localize in the mitochondrial matrix and improve energy production [14]. Several strategies have been developed to reduce Mcl-1 expression within tumor cells. One of these is the modulation of glycolytic metabolism, which regulates Bcl-2 family members expression, especially Mcl-1, but also sensitizes tumor cells to apoptosis through the modulation of the main energy sensor of the cell, the AMPK/mTOR pathway [15–19]. We recently established that reducing the food intake of the mice by 25% (caloric restriction, CR) reduces Mcl-1 expression and sensitize lymphoma-bearing mice to BH3-mimetics [20]. We therefore decided to investigate if a specific macronutrient of the food could impact on Mcl-1 expression. We fed mice bearing non Hodgkin B cell lymphoma with an isocaloric low carbohydrate (CHO) or an isocaloric low protein (PROT) diet and analyzed their response to targeted chemotherapy. Our work indicates that lowering CHO intake represents an efficient way to downregulate Mcl-1 via an AMPK/mTOR dependent control of its translation.

## RESULTS

### Low carbohydrate diet reduces Mcl-1 expression

**Figure 1:**
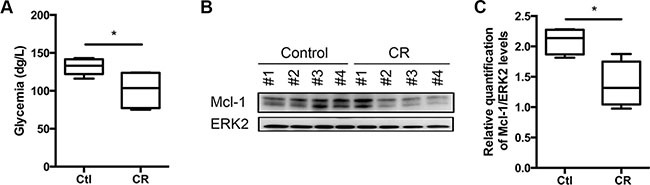
Caloric Restriction decreases Mcl-1 protein levels WT C57BL/6 syngeneic mice were injected intravenously with Eμ-Myc lymphoma cells and fed ad libitum (Ctl, control) or under CR conditions for 5 days (5 mice per group). (**A**) Glycemia was measured after 5 days of dietary study. (**B**) Lymph nodes bearing lymphoma were harvested from 5 independent mice after 5 days under CR or ad libitum feeding (control) and lysates were prepared. Mcl-1 expression was analyzed by immunoblots. (**C**) Average quantification of Mcl-1 compared with ERK2 levels (used as a loading control) from samples. *P < 0.05.

Using an *in vivo* model of Myc lymphoma-mice [21], we addressed the role of caloric intake and specific nutrients in the resistance to BH3-mimetics. Myc lymphoma-bearing mice are especially well suited model to address this question. First, Eμ-*Myc* transgenic mice overexpress the c-*Myc* oncogene in the B cell lineage and develop pre-B and B-cell lymphoma with associated leukemia by several months of age [21]. Secondly, genetics and histopathology of those mice resemble human non-Hodgkin's lymphomas. Thirdly, we chose the Eμ-*Myc* model as it was largely proven to be a very robust system to characterize *in vivo* response to anti-cancer drugs [22] and to decipher the key role-played by the Bcl-2 family members in this setting [23, 24]. We therefore investigated how caloric intake may modulate the expression of this family of proteins. To this end, syngeneic C57BL/6 mice were intravenously injected with Eμ-*Myc* primary cells. Four days later, lymphoma-bearing mice were fed either *ad libitum* (control) or in CR conditions, which consists in a 25% reduction of caloric intake, in accordance with our recent work [20], we showed that a global reduction of caloric intake by 25% for 5 days was sufficient to reduce the glycemia of the mice (Figure 1A) and to decrease Mcl-1 expression by 50%(Figure 1B, 1C). On note, the mice weight was not modulated over the time of the experiment (not shown).

**Figure 2:**
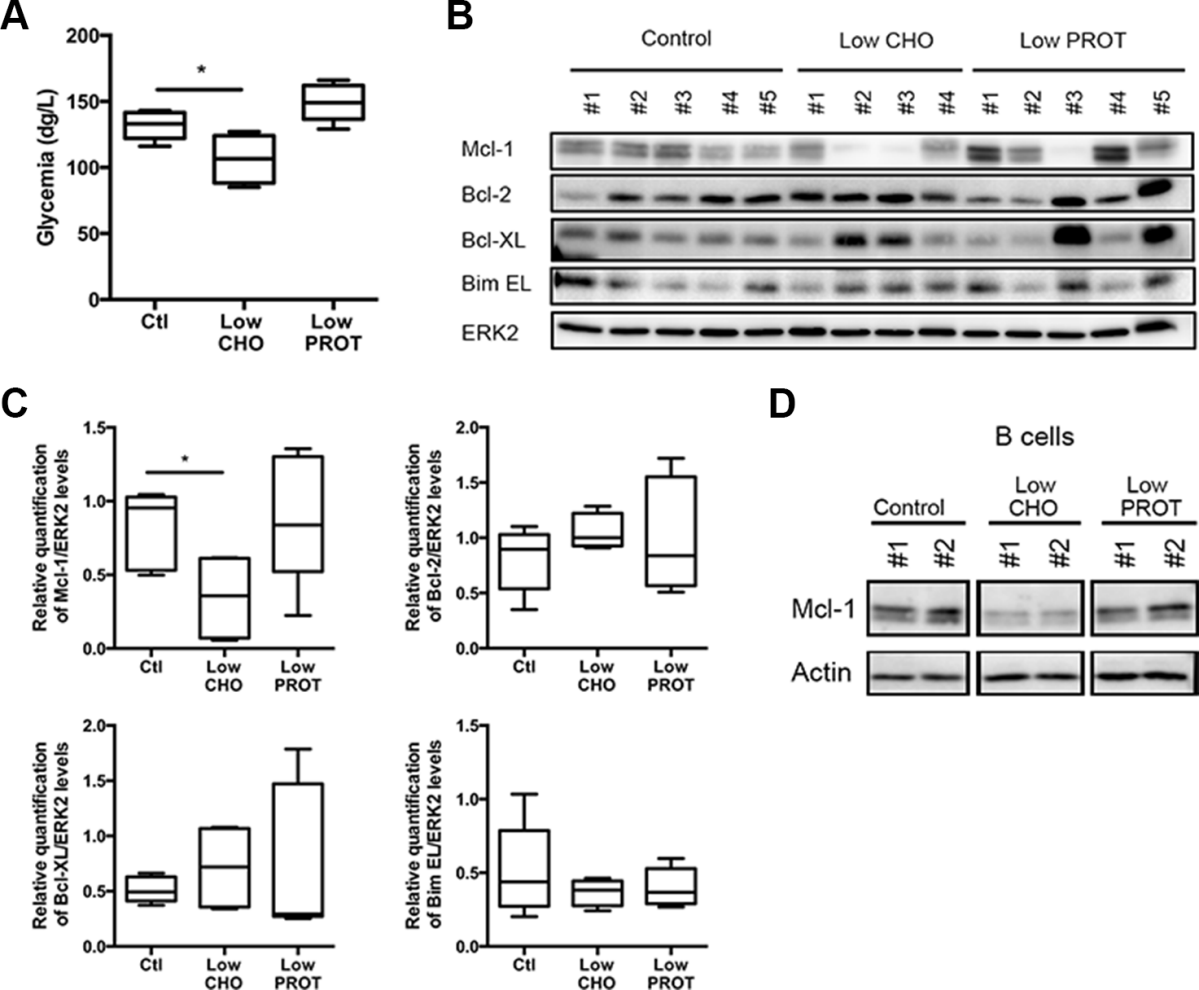
Low carbohydrate Intake affects Mcl-1 expression levels WT C57BL/6 syngeneic mice were injected intravenously with Eμ-Myc lymphoma cells and fed *ad libitum* with Ctl, low CHO and low PROT diets (Ctl, *n* = 5; low CHO, *n* = 4; low PROT, *n* = 5). (**A**) Glycemia was measured after 5 days of dietary study. *P < 0.05. (**B**) Lymph nodes bearing lymphoma were harvested from mice after 5 days of *ad libitum* feeding with Ctl, low CHO and low PROT diets and lysates were prepared. Expression of Bcl-2 family members was analyzed by immunoblots. (**C**) Average quantification of Mcl-1, Bcl-2, Bcl-XL, and Bim EL levels compared with ERK2 levels (used as a loading control). *P < 0.05. (**D**) WT C57BL/6 syngeneic mice were injected intravenously with Eμ-*Myc* lymphoma cells and fed ad libitum with Ctl, low CHO and low PROT diets for 5 days. Lymph nodes bearing lymphoma were harvested from mice and B cells were sorted (Ctl, *n* = 2; low CHO, *n* = 2; low PROT, *n* = 2). Mcl-1 protein levels were analyzed by immunoblot. Actin was used as a loading control.

We then sought to determine which macronutrient was involved in the decrease of Mcl-1 protein expression. We therefore derived from the control diet two additional custom diets that contain 25% less carbohydrates (hereafter Low CHO diet) or 25% less proteins (Low PROT diet). It is worth noting that all the diets are isocaloric (see material and methods section for details). Syngeneic C57BL/6 mice were intravenously injected with Eμ-*Myc* primary cells and a few days later lymphoma-bearing mice were fed *at libitum* with one of these diets for 5 days. Of note, there was no significant difference in the weight of the mice fed with the different diets over the course of the study (not shown). We observed that only the Low CHO diet significantly reduced glycemia levels (Figure 2A). Upon sacrifice, we confirmed by flow cytometry that the CD3 and CD19 populations in the lymph nodes and spleens were similar and therefore comparable among mice (Supplementary Figure S1A, S1B). We therefore analyzed the expression levels of the main Bcl-2 family members within the lymph nodes. Only a few days of feeding the mice with the Low CHO diet repeatedly led to a reduction of Mcl-1 expression (Figure 2B, 2C) by more than 50%. It is worth mentioning that other Bcl-2 family members analyzed (i.e. Bcl-2, Bcl-xL and Bim EL) were not significantly modulated by this diet (Figure 2B, 2C). In order to determine whether the decrease in Mcl-1 protein expression represents a decrease in lymphoma B cells, lysates from sorted B cells isolated from lymphoma-bearing spleens were analyzed by Western blot. B cells showed a uniformly low level of expression of Mcl-1 under Low CHO conditions, while this was not observed under control or Low PROT conditions (Figure 2D).

Altogether our data indicate that carbohydrates are the main macronutrients involved in the diet-induced reduction of Mcl-1 expression in B cells of lymphoma-bearing mice.

### Diet-induced decrease of Mcl-1 occurs through a reduction of its translation in an AMPK-mTOR dependent manner

**Figure 3:**
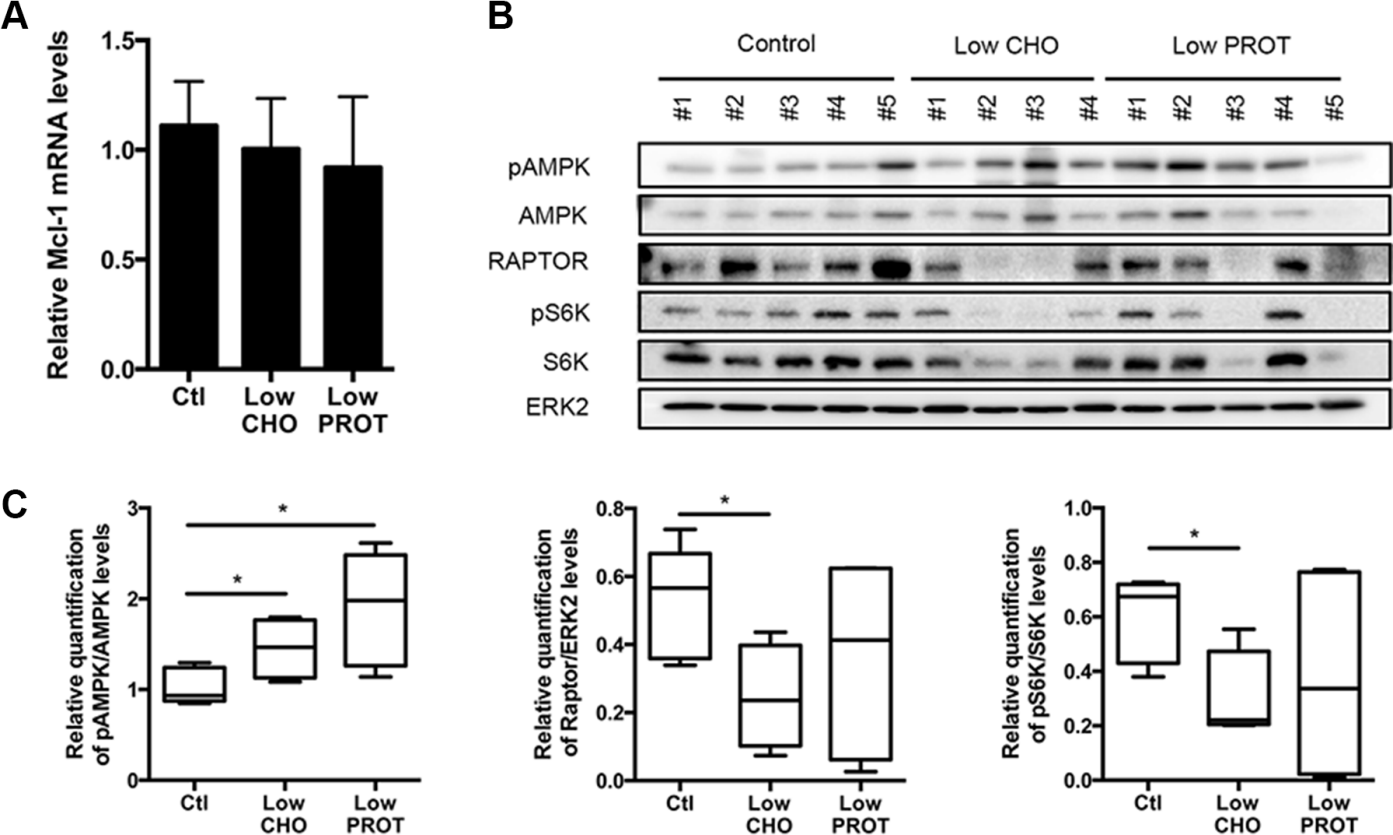
Low carbohydrate intake inhibits mTOR signaling Lymph nodes bearing lymphoma were harvested after 5 days of *ad libitum* feeding with Ctl, low CHO and low PROT diets and (**A**) Mcl-1 mRNA levels were measured by real-time qPCR. (Ctl, *n* = 4; low CHO, *n* = 4; low PROT, *n* = 4) (**B**) Expression of proteins from the AMPK/mTOR pathway was analyzed by immunoblots. Average quantification of pAMPK, Raptor and pS6K compared with ERK2 levels (used as a loading control) or total protein levels. (Ctl, *n* = 5; Low CHO, *n* = 4; Low PROT, *n* = 5). *P < 0.05.

In order to determine how Mcl-1 expression was reduced upon Low CHO diet, we first measured its mRNA expression. As previously, lymphoma-bearing mice were fed with a Low CHO or a Low PROT diet for 5 days and Mcl-1 mRNA levels from the lymph nodes were analyzed by real time quantitative PCR. We did not observe any significant difference between the diets (Figure 3A), suggesting that Mcl-1 regulation upon dietary modulation occurs at the post-transcriptional level.

Among the Bcl-2 family members, Mcl-1 has the particularity of being a protein with a short half-life. We and others have reported that an inhibition of the AMPK/mTOR pathway results in the inhibition of protein translation and subsequent decrease of Mcl-1 expression [18–20, 25, 26]. It is well established that upon reduction of nutrient availability, cells will reduce protein translation, as it is one of the most ATP-demanding process in the cell. This process is mainly regulated by the AMPK/mTOR signaling pathway. We observed that AMPK was activated in the lymph nodes of lymphoma-bearing mice that were fed during 5 days with the Low CHO and Low PROT diets. We also observed an inhibition of the mTOR pathway in the lymph nodes of mice fed with the Low CHO diet as judged by the reduction in RAPTOR expression along with reduced level of phosphorylated S6, one of the mTOR targets (Figure 3B, 3C).

**Figure 4:**
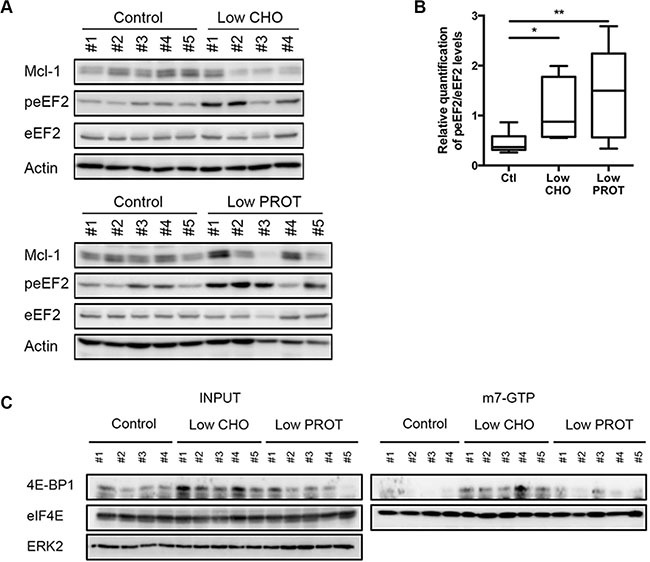
Low carbohydrate intake inhibits protein translation (**A**) Lymph nodes were harvested from mice after 5 days of *ad libitum* feeding with Ctl, low CHO and low PROT diets and lysates were prepared (Ctl, *n* = 5; low CHO, *n* = 4; low PROT, *n* = 5). peEF2 expression was analyzed by immunoblot and normalized to the total protein. Actin was used as a loading control. (**B**) Average quantification of peEF2 normalized to total protein levels. (**C**) Lymphocytes were harvested from mice after 5 days of *ad libitum* feeding with Ctl, low CHO and low PROT diets and cap-dependent translation was assessed by m^7^-GTP pull down. The association between 4E-BP1 and eIF4E was revealed by immunoblot. Association of the inhibitory subunit 4E-BP1 with eIF4E indicates an inhibition of translation. Expression of indicated proteins was analyzed by immunoblots. *P < 0.05; **P < 0.01.

Eukaryotic Translation Elongation Factor 2 (eEF2) is a key regulator of protein translation that is controlled by AMPK activation and/or mTOR inhibition [27]. Indeed, its phosphorylation leads to its inactivation resulting in a block in protein translation. We observed *in vivo* that the Low CHO and the Low PROT diet resulted in the inactivation of eEF2, which was associated with a reduction in Mcl-1 expression (Figure 4A, 4B). To further investigate cap-dependent protein translation inhibition *in vivo*, we performed m^7^-GTP pull down in lymphocytes of sick mice. eIF4E and associated factors were isolated with m^7^-GTP (guanosine 50-triphosphate)- sepharose beads, which mimics the mRNA cap structure. As shown in Figure 4C, the amount of inhibitory 4E-BP1 associated with eIF4E increased dramatically in mice fed with a Low CHO diet, indicating an inhibition of translation rates.

Taken together, these results indicate that the Mcl-1 decrease observed *in vivo* in lymphoma-bearing mice fed with a Low CHO diet is mediated, at least in part, by the AMPK/mTOR control of its translation.

### Low carbohydrate diet sensitizes lymphoma-bearing mice to ABT-737-treatment

We established that a 25% reduction in CHO was sufficient to significantly reduce Mcl-1 expression in lymphoma cells (Figures 2, 4). Eμ-*Myc* cells are known to be resistant to ABT-737 mainly because they express high Mcl-1 levels, which is poorly targeted by this BH3-mimetic. We tried to mimic the effects of macronutrient modulation *in vitro*, where Eμ-*Myc* and HeLa cells were resistant to ABT-737 but were sensitized to it when glycolysis was inhibited or glucose in the medium was modulated. *Myc*-expressing premalignant and neoplastic B cells are highly sensitive to amino acid (AA) starvation, inducing extensive cell death *in vitro* and making it hard to analyze its effect on ABT-737 resistance (Supplementary Figure S2A). In the case of HeLa cells, we observed sensitization to ABT-737 by glycolysis inhibition and modulation, but not in AA modulation and deprivation conditions. The sensitization to ABT-737 was lost when Mcl-1 was overexpressed in these cells (Supplementary Figure S2B).

**Figure 5:**
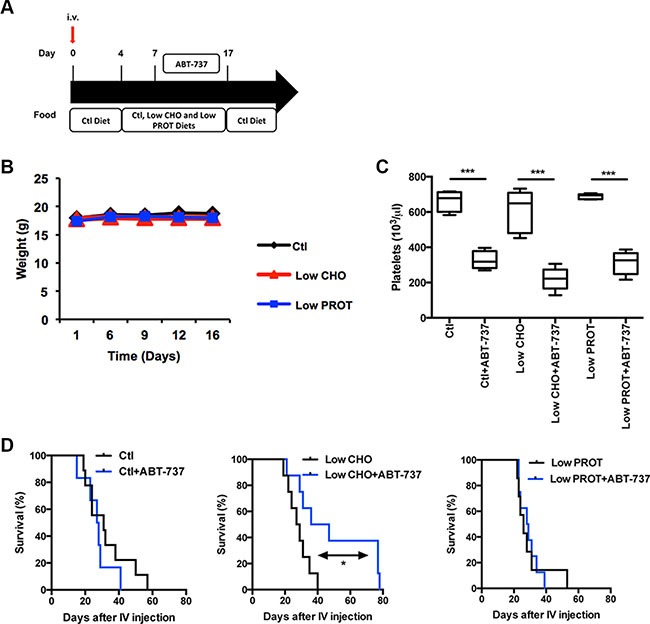
Low carbohydrate intake increases tumor free survival of mice treated with ABT-737 (**A**) Design of the experiment: Syngeneic C57BL/6 mice were intravenously injected with Eμ-Myc lymphoma cells and fed *ad libitum* with Ctl, low CHO and low PROT diets for 14 days. 7 days after intravenous injection, mice were treated or not for 10 days with 75 mg/kg ABT-737. Subsequently, all mice were fed ad libitum with the Ctl Diet until the time of ethical euthanasia. (**B**) Average weight of the mice that were fed for 14 days with Ctl, low CHO and low PROT diets (10 mice per group). (**C**) Numbers of platelets were measured in each group 9 days after the beginning of ABT-737 treatment (10 mice per group). (**D**) Tumor free survival of the mice are indicated for each group (Ctl *n* = 9, Ctl+ABT-737 *n* = 6, Low CHO *n* = 8, Low CHO+ABT-737 *n* = 8, Low PROT *n* = 7, Low PROT+ABT-737 *n* = 8). *P < 0.05, ***P < 0.005. When not mentioned, differences are not significant.

We therefore investigated if the modulation of macronutrient intake was sufficient to sensitize lymphoma-bearing mice to this targeted chemotherapy. Lymphoma-bearing mice were fed *ad libitum* with the indicated diets. 3 days later, mice were intraperitoneally injected on a daily basis with 75 mg/kg ABT-737 or vehicle for 10 days (Figure 5A). At the end of the treatment, all the mice were fed with the control diet to limit the impact of those diets on tumor development. We verified that neither the type of food (Figure 5B), nor ABT-737 treatment impacted on the weight of the mice (not shown). We validated that ABT-737 treatment was effective as it led to thrombocytopenia (Figure 5C), as previously described [28]. It is worth noting that thrombocytopenia was equivalent regardless of the type of diet, consistent with Mcl-1 not playing an essential role in platelet survival [29]. Consistent with this pre-clinical model, ABT-737 treatment did not increase mice survival when animals were fed with the control diet. We observed that the different diets did not significantly modulate the overall survival of the mice in absence of chemotherapy (Ctl vs. Low CHO: *P* = 0,3546; Ctl vs. Low PROT: *P* = 0,5008; Low CHO vs. Low PROT: *P* = 0,9596). On the opposite, the combination of a Low CHO but not a Low PROT diet with ABT-737 doubled the overall survival of the mice (increasing the overall survival of the mice from 40 days in Low CHO conditions to 79 days in Low CHO + ABT-737 conditions, Ctl vs. Ctl+ABT-737: *P* = 0,3179; Low CHO vs. Low CHO+ABT-737: *P* = 0,0487, Low PROT vs. Low PROT+ABT-737 *P* = 0,9187, Figure 5D). This effect was observed regardless of the Eμ-*Myc* clone used (See Supplementary Figure S3).

Overall, our results indicate that lowering carbohydrates but not protein intake is sufficient to reduce Mcl-1 expression and to sensitize lymphomas in mice to treatment with ABT-737.

## DISCUSSION

Overexpression of Mcl-1 has been observed in several human cancers [30] and is implicated in resistance to anti-cancer therapeutics. Mcl-1 specific inhibitors have been recently developed but their use as potential therapeutic treatment is still under investigation [12]. While we obtain clinical validation of such inhibitors, several attempts to reduce Mcl-1 expression have been proposed, such as the modulation of cellular metabolism (for review [13]). Here we report on the ability of a Low CHO diet to reduce Mcl-1 protein expression and to sensitize lymphoma cells to BH3-mimetics.

While reducing food intake (caloric restriction) is a very efficient way to reduce Mcl-1 expression and to sensitize lymphoma cells to targeted therapies [20], the clinical relevance of such approach might be limited by the general condition of the patient. Indeed, cachexia that is characterized by a massive loss of total body mass, anorexia, general inflammation and pronounced muscle-wasting results in a drastic decrease of the quality of life and is associated with a poor prognosis [31]. Up to 80% of cancer patients will face cachexia and it is therefore not appropriate to reduce caloric intake in most of them. To circumvent this limitation and to benefit from the positive effects brought by CR, several options are nevertheless available. One option would be to use the so-called caloric restriction mimetics (CRm). CRm are molecules that lead to one or several physiological changes induced by CR including reduction in blood glucose, insulin, and triglycerides without modulating food intake. In this line several molecules such as metformin, resveratrol or mTOR inhibitors (rapalogues) could represent interesting options (recently reviewed in [4, 32]). We and others have described that the use of such CRm could represent a possible way to limit Mcl-1 expression *in vivo* and to sensitize tumor-bearing mice to BH3-mimetics-induced death [15–19]. However the exact mechanism on how those compounds are working at the molecular level and what are the efficient doses required *in vivo* to mimic the effects of CR in patients is still highly debated in the field [33].

Another option would be to identify which macro-component of the diet is responsible for the observed decrease of Mcl-1 upon CR in order to benefit of this effect without reducing general caloric intake. For that matter, as we established that a 25% reduction of caloric intake was sufficient to reduce Mcl-1 expression ([20] and Figure 1), we generated custom diets presenting a 25% reduction of either carbohydrates (CHO) or protein (PROT). In order to limit the impact of those diets on tumor development and in order to analyze the response to targeted therapy, we fed the mice with the custom diets only during the course of the treatment with ABT-737 (See Figure 5A).

B-cells, circulating and those residing in the lymph nodes, receive survival, and proliferative signals from B-cell receptor signaling through the PI3K/AKT/mTOR pathway [34]. mTOR is activated by phosphoinositide-3 kinase (PI3K)/Akt signaling in the presence of nutrients and growth factors, and inhibited by AMPK in the setting of energy deprivation. Additionally, it has been described that mTOR pathway regulates Mcl-1 by 4EBP1/mTORC1-dependent translation [19, 20, 25]. We observed that feeding lymphoma-bearing mice with a Low CHO diet resulted in AMPK activation (Figure 3), mTOR and eEF2 inhibition (Figures 3 and 4) indicating a block in protein translation. This block was further supported using m^7^-GTP binding experiments (Figure 4C). As Mcl-1 has a short half-life, even a partial protein translation inhibition is sufficient to reduce its protein expression. Very importantly, we observed that the reduction in Mcl-1 expression upon a Low CHO diet was sufficient to significantly sensitize lymphoma-bearing mice to ABT-737 induced death (Figure 5D).

We noticed that the effect of a Low PROT diet over Mcl-1 expression levels (Figure 2B, 2C) was very variable among experiments and not sufficient to sensitize lymphoma-bearing mice to ABT-737 treatment (Figure 5D). It has been suggested that PROT restriction primarily impacts the IGF-1 signaling pathway and that its impact on insulin levels, glycemia, ketone bodies, and free fatty acids relies on the conversion of amino acids to glucose via gluconeogenesis [35]. Therefore, changes in glucose production following a Low PROT diet are less pronounced when compared to a Low CHO diet [36]. We therefore conclude that a Low CHO diet is a more robust and efficient way to decrease Mcl-1 expression and it is very likely to be the main component, albeit not the unique involved in this effect. This conclusion is also supported by the widely described effects of glycolytic inhibitors on Mcl-1 expression and on the sensitization toward ABT-737 [4, 15–17, 20]. Altogether our results indicate that lowing circulating glucose is a key event in the decrease of Mcl-1 expression. In this sense, our results are encouraging as there are several ongoing clinical trials investigating low CHO diets in combination with conventional therapies for cancer treatment [35].

Overall, we established that a short-term reduction of carbohydrate intake represents an innovative and safe way to reduce the expression of Mcl-1, a very important oncogenic protein in lymphoma cells, thereby sensitizing them to targeted chemotherapy.

## MATERIALS AND METHODS

### CR and macronutrient modulation experiments

All animal experiments were performed according to the guidelines of the Institutional Animal Care and Use Committee and of the regional ethics committee (approval reference PEA-232). Eμ-Myc/wild-type (WT) mice were originally obtained from the Jackson Laboratory. WT syngeneic C57BL/6 mice were intravenously (i.v.) injected with 0.1 × 10^6^ Eμ-Myc cells and were then fed *ad libitum* with a Control, low Carbohydrates (Low CHO) or low Protein diet (Low PROT, see Table 1) custom generated by Envigo or in CR mode (75% of normal dose is 2.25 g per day per mouse). Seven days after intravenous injection of lymphoma cells, mice were intraperitoneally (I.P.) injected daily for 10 days with vehicle or ABT-737 (75 mg/kg). At the end of the ABT-737 treatment, all mice were fed *ad libitum* with control diet. Mice were monitored for lymphoma development and systemic signs of illness: apathy, breathing problems, precipitous weight loss, and limited ability to reach food or water. Animals were euthanized as soon as they showed any signs of illness. After 5 days of diet intake, glycemia was measured after a few hours of fasting by using a freestyle Optium blood glucose monitoring device. The number of Platelets was measured 1 day before the end of the ABT-737 treatment by using a Hemavet 950FS (Drew Scientific, Inc., Le Rheu, France). Mice survival was measured from the day of i.v. injection of Eμ-*Myc* mice to the death of the mice.

**Table 1:** Macronutrient breakdown of diets used

Diet	Control	Low CHO	Low PROT
**Carbohydrates**	70,9	54,0	73,6
**Proteins**	19,5	26,9	14,8
**Lipids**	9,6	19,2	11,6

Note: Values are given in % kcal.

### *In vitro* treatments

For nutrient modulation experiments, cells were either treated for 20 hours with 2DG (25 mM for HeLa cells and 600 μM for Eμ-*Myc* cells), cultured in a medium without glucose, with ¼ the normal concentration of amino acids or in a medium with no amino acids. In all these conditions cells were cultured in the presence or absence of 10 μM ABT-737. The empty vector and pSPEC Mcl-1 plasmid were a kind gift of Dr. Maurer (Freiburg, Germany).

### Western blot analysis

Lymph nodes were collected and lysed using a Precellys 24 homogenizer (3 × 30 s, 6500 × g) in buffer A :10 mM HEPES (pH 7.4), 150 mM NaCl, 5 mM EDTA, 1% NP40, 10 μg/mL aprotinin, 1 mM phenylmethylsulfonyl fluoride (PMSF), 10 mM leupeptin. CD19+ cells were isolated from the spleens of lymphoma-bearing mice using autoMACS (Miltenyi Biotec). CD19+ sorted cells and other cell lines were lysed in in Laemmli sample buffer. Proteins were immunoblotted with the indicated antibodies. Immunoblots were revealed (FUJIFILM LAS4000, France) using enhanced chemiluminescence detection kit (Pierce) and quantification was made using ImageJ software. The antibody Anti Mcl-1 was obtained from Rockland (Gilbertsville, PA, USA). Anti Bcl-xL, Bcl-2, Bim, pAMPK (Thr172), AMPK, RAPTOR, pS6K(Thr389), S6K, peEF2 (Thr56), eEF2, 4E-BP1, eIF4E and were purchased from Cell Signaling Technology^®^ (Danvers, MA, USA). Anti-ERK2 and Actin were purchased from Santa Cruz (Santa Cruz, CA, USA).

### Quantitative reverse transcription-PCR analysis

Total RNA was isolated from lymphoma cells using the RNeasy Micro Kit (Qiagen, Paris, France) according to the manufacturer's protocol. After reverse transcription-PCR, the relative mRNA expression level of mouse Mcl-1 was obtained by real-time quantification PCR, using the TaqMan PCR Master Mix (Eurogentec, Seraing, Belgium) and TaqMan assay primer set (Applied Biosystems, Foster City, CA, USA) on the 7500 Fast and the Step One (Applied Biosystems) according to the manufacturer's instructions. The housekeeping gene RPLP0 was used as a control for RNA quality, and used for normalization.

### 7-Methyl-GTP cap binding assay

Spleens from mice fed *ad libitum* with a Control, Low CHO or Low PROT diet were lysed in 400 μl of 20 mM Tris pH 7.5, 100 mM KCl, 20 mM β-glycerophosphate, 1 mm dithiothreitol, 250 μM Na_3_VO_4_, 10 mM NaF, 1 mM EDTA, 1 mM ethylene glycol tetraacetic acid, 1 mM phenylmethylsulfonyl fluoride and lysed using a Precellys 24 homogenizer (2 × 30 s, 6500 × g). After centrifugation (13000 g—10 min—4°C), 450 μg of protein was applied to 50 μl of m^7^-GTP-sepharose 4B beads (Jena Bioscience, Jena, Germany) and incubated for 2 hours at 4°C. The beads were washed and then boiled in Laemmli sample buffer. After SDS–PAGE resolution, the association of 4E-BP1 with eIF4E was detected by western blot.

### Cell viability and flow cytometry

Flow Cytometry (MACS-Quant Analyzer Miltenyi Biotec) was used to analyze cell viability by looking at plasma membrane permeabilization of cells using 4′,6-diamidino-2-phenylindole (DAPI) staining. Cell viability was measured as the percentage of DAPI-negative cell population. Lymphocyte subpopulations from lymphoma-bearing lymph nodes and spleens were assessed by flow cytometry using antibodies against CD3 and CD19 (BD Bioscience, Franklin Lakes, NJ, USA).

### Statistics

The data are expressed as mean ± standard deviation. Differences in calculated means between groups were assessed by two-sided Student *t* tests. For experiments involving more than two groups, differences in the calculated mean values between the groups were assessed by one-way analysis of variance, followed by a Fisher test, and in cases in which significant differences were detected, a Tukey honestly significant difference test was used. Kaplan-Meier survival analyses were performed, and survival curves were compared by using log-rank tests. For *in vitro* experiments, differences in the calculated mean values (binomial law) between the groups were assessed by two-way analysis of variance followed by a χ^2^ test. A *P* value less than 0.05 was considered significant.

## SUPPLEMENTARY MATERIALS FIGURES


