# Supplementary material for: Low carbohydrate diet prevents Mcl-1-mediated resistance to BH3-mimetics
Source: Oncotarget. 2016 Sep 28;7(45):73270–9. doi: 10.18632/oncotarget.12309 (PMC5341978; doi:10.18632/oncotarget.12309)
Supplement: Supplementary file 1 [file oncotarget-07-73270-s001.pdf]

Low carbohydrate diet prevents Mcl-1-mediated resistance to BH3-mimetics

Supplementary Materials

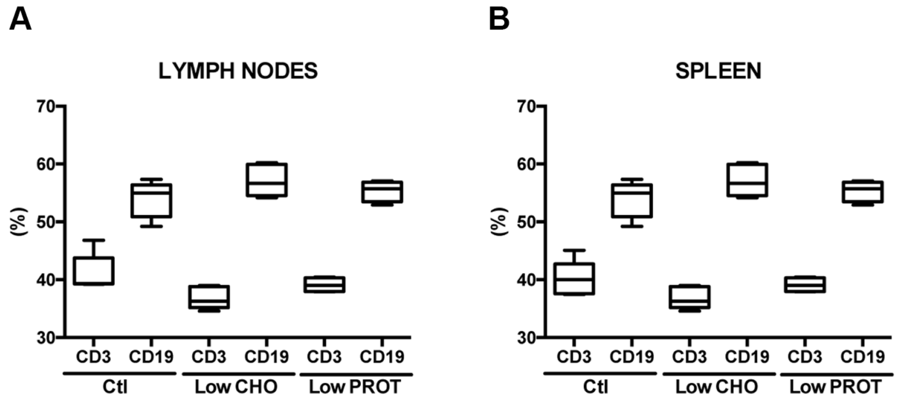

Supplementary Figure S1: Lymphocyte subpopulations in lymphoma-bearing lymph nodes and spleens. WT C57BL/6 syngeneic mice were injected intravenously with Eμ-Myc lymphoma cells and fed ad libitum with Ctl, low CHO and low PROT diets (Ctl, *n* = 5; low CHO, *n* = 5; low PROT, *n* = 4) for 5 days. (**A**) The proportion of T cells (CD3+) and B cells (CD19+) was analyzed by flow cytometry in lymph nodes and (**B**) spleens of lymphoma bearing mice.

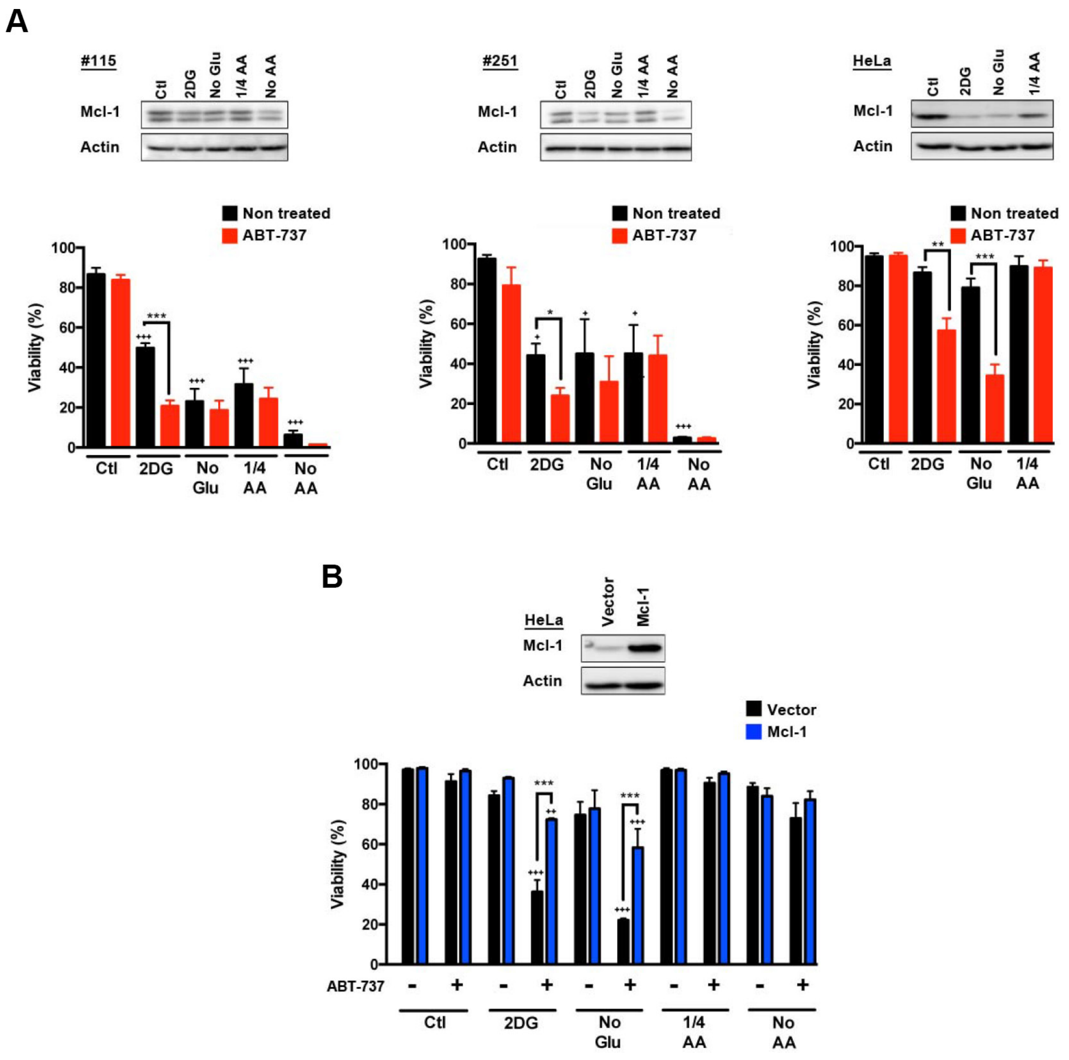

Supplementary Figure S2: Glucose modulation and inhibition sensitizes cells to ABT-737 *in vitro*. (**A**) Cells were incubated with 2DG and the indicated amount of glucose (Glu) or amino acids (AA) in the presence (red bars) or absence (black bars) of ABT-737 for 20 hours. The levels of Mcl-1 were analyzed by immunoblot. Actin was used as a loading control. Cell death was determined by flow cytometry using DAPI. $^{*}P < 0.05$; $^{**}P < 0.01$; $^{**}P < 0{,}005$. (**B**) HeLa cells were stably transduced with an empty vector (black bars) or an Mcl-1 overexpressing vector (blue bars). Cells were incubated with 2DG or with the indicated amount of glucose (Glu) and amino acids (AA) in the presence (red bars) or absence (black bars) of ABT-737 for 20 hours. Cell death was determined by flow cytometry using DAPI. $^{++}P < 0.01$; $^{+++}P < 0.005$ compared to untreated control cells. $^{*}P < 0.05$; $^{***}P < 0.005$ compared to treated cells. When not mentioned, differences are not significant.

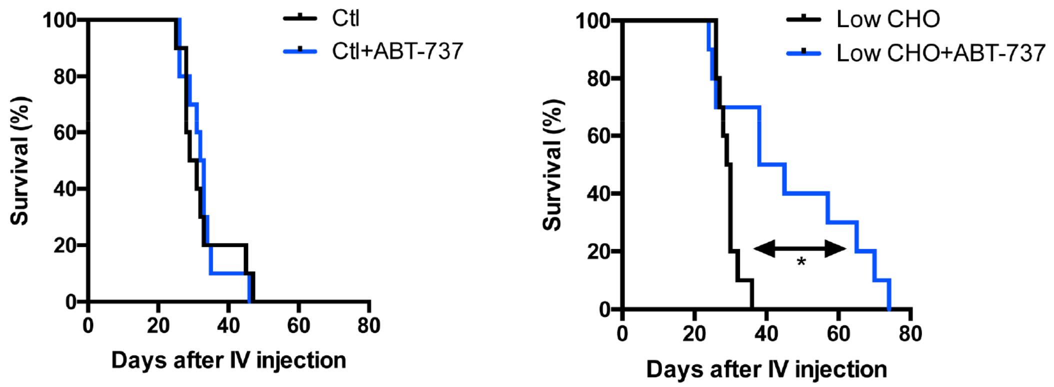

Supplementary Figure S3: Low CHO diet increases tumor free survival of lymphoma bearing mice regardless of the Eµ-Myc clone used. Experiment was conducted as in Figure 5 using an independent Eµ-Myc clone. Design of the experiment: Syngeneic C57BL/6 mice were intravenously injected with Eµ-Myc lymphoma cells and fed ad libitum with Ctl, low CHO and low PROT diets for 14 days. 7 days after intravenous injection, mice were treated or not for 10 days with 75 mg/kg ABT-737. Subsequently, all mice were fed ad libitum with the Ctl Diet until the time of ethical euthanasia. Tumor free survival of the mice are indicated for each group (Ctl $n = 10$, Ctl+ABT-737 $n = 10$, Low CHO $n = 10$, Low CHO+ABT-737 $n = 10$). *$P < 0.05$. When not mentioned, differences are not significant.
